# Complex Peripheral Arterial Disease in a Case of Multilevel Lesions Treated With Sequential Angioplasty

**DOI:** 10.7759/cureus.60982

**Published:** 2024-05-24

**Authors:** Ishiqua V Patil, Prerit Sharma, Gajanan Pisulkar, Ankur Salwan, Maharshi Patel

**Affiliations:** 1 Hospital Administration, Datta Meghe Institute of Higher Education and Research, Wardha, IND; 2 Interventional Radiology, Datta Meghe Institute of Higher Education and Research, Wardha, IND; 3 Orthopedic Surgery, Jawaharlal Nehru Medical College, Datta Meghe Institute of Higher Education and Research, Wardha, IND; 4 General Internal Medicine, Jawaharlal Nehru Medical College, Datta Meghe Institute of Higher Education and Research, Wardha, IND

**Keywords:** perfusion, occlusions, claudication, revascularization, multilevel lesions

## Abstract

Peripheral artery disease (PAD) is the buildup of calcium and fatty deposits in the arterial walls (atherosclerosis). This is an important clinical issue, specifically in cases with multilevel lesions. A patient underwent sequential angioplasty treatment for major PAD, which was characterized by multilevel lesions affecting both the infrapopliteal arteries. The proximal vessels and infrapopliteal vessels are mostly observed to be affected by PAD, thus the patient likely has PAD localized to the lower leg. In the femoropopliteal segment, lower extremity artery or aortic atherosclerotic occlusive disease can lead to significant outcomes. Severe claudication and pain during rest in both legs were observed in a patient with a history of hypertension and diabetes mellitus. With an angiography, the superficial femoral, popliteal, and tibial arteries have been shown to have major stenoses and occlusions. A progressive treatment was used because of the complexity of the lesions initiating with endovascular revascularization of the superficial femoral artery. The popliteal and tibial arteries were then repaired with angioplasty and stent placement. After the treatment, the patient's symptoms significantly improved, including elimination of their rest discomfort and claudication. Measurements of the ankle-brachial index (ABI) indicated that the affected limbs' perfusion was refined. Six months later, a follow-up angiography revealed intact vessels with no restenosis. This case report shows the successful outcome of recurrent angioplasty in curing complicated multilevel PAD, giving symptomatic relief and maintaining limb perfusion. This research is required to assess the long-term outcomes and longevity of this kind of treatment in patient populations that are comparable to others.

## Introduction

Peripheral artery disease (PAD) is a prevalent and debilitating condition characterized by atherosclerotic narrowing or occlusion of the arteries supplying the extremities [1]. PAD affects the worldwide health of the population by presenting symptoms in the beginning and ending with severe pain that worsens with time [2]. Early detection can help individuals with nonsurgical treatments. This is most commonly seen in senior citizens who have associated problems like hypertension and diabetes mellitus [3]. Endovascular methods of treatment, specifically angioplasty, have become a key component of the treatment regime for PAD patients, offering less invasive options to the more prevalent surgical revascularization. A difficult case of PAD in a 67-year-old patient who had a history of hypertension and diabetes mellitus is presented[4]. The patient showed severe claudication and pain in both lower limbs, which are signs of severe arterial insufficiency and limb ischemia. Diagnostic angiography revealed multilevel lesions affecting the superficial femoral, popliteal, and tibial arteries, indicating the complex nature of the vascular illness. Revascularization would have to be done through various stages due to the type and severity of the lesions. The preferred treatment method was sequential angioplasty, an endovascular procedure that treats the artery one segment at a time. This method reduces the danger of consequences while increasing success due to its focused treatment options and full lesion assessment [5,6]. The superficial femoral artery's proximal lesions were the primary target of this first stage of revascularization. Conventional endovascular techniques, like balloon angioplasty and placement of drug-eluting stents, effectively restored blood flow in the superficial femoral artery. Later, angiography confirmed the removal of large stenoses and improved turbulent and arterial flow [7]. Also, we go into the reasons behind the sequential angioplasty procedure and how it helps to achieve the best results.

## Case presentation

A 67-year-old male patient was referred to Shalinitai Meghe Hospital, Wardha. The patient came with a history of diabetes mellitus and hypertension. The patient claimed severe bilateral leg pain that had been worse over the past few weeks, especially after physical tasks. He described the pain and having cramps in both calves that made it very difficult to walk even a few steps also his pain worsened at night while sleeping. On examination, the patient showed signs of PAD while being at rest. There was bilateral pallor in the lower limbs. Both sides had reduced peripheral pulses, and there were no dorsalis pedis or posterior tibial pulses. In feet, the patient also showed decreased sensitivity to light touch and pinprick sensation, which are signs of peripheral neuropathy. The patient had no symptoms of paresthesia, pallor, or acute limb ischemia. The patient's clinical presentation and further diagnostic examination were conducted, including non-invasive vascular inquiries and angiography. Ankle-brachial index (ABI) measures found significant arterial insufficiency; bilateral ABI values of 0.6 were suggestive of severe PAD that caused ischemic rest pain. The presence of multiple arterial lesions, including the superficial femoral, popliteal, and tibial arteries with a range of stenosis and occlusion, was confirmed by a follow-up diagnostic angiography. Considering the complex nature of the patient's arterial lesions and his severe symptom burden, vascular surgeons, interventional cardiologists, and radiologists came together to decide on the best approach to action.

Procedure

**Figure 1:**
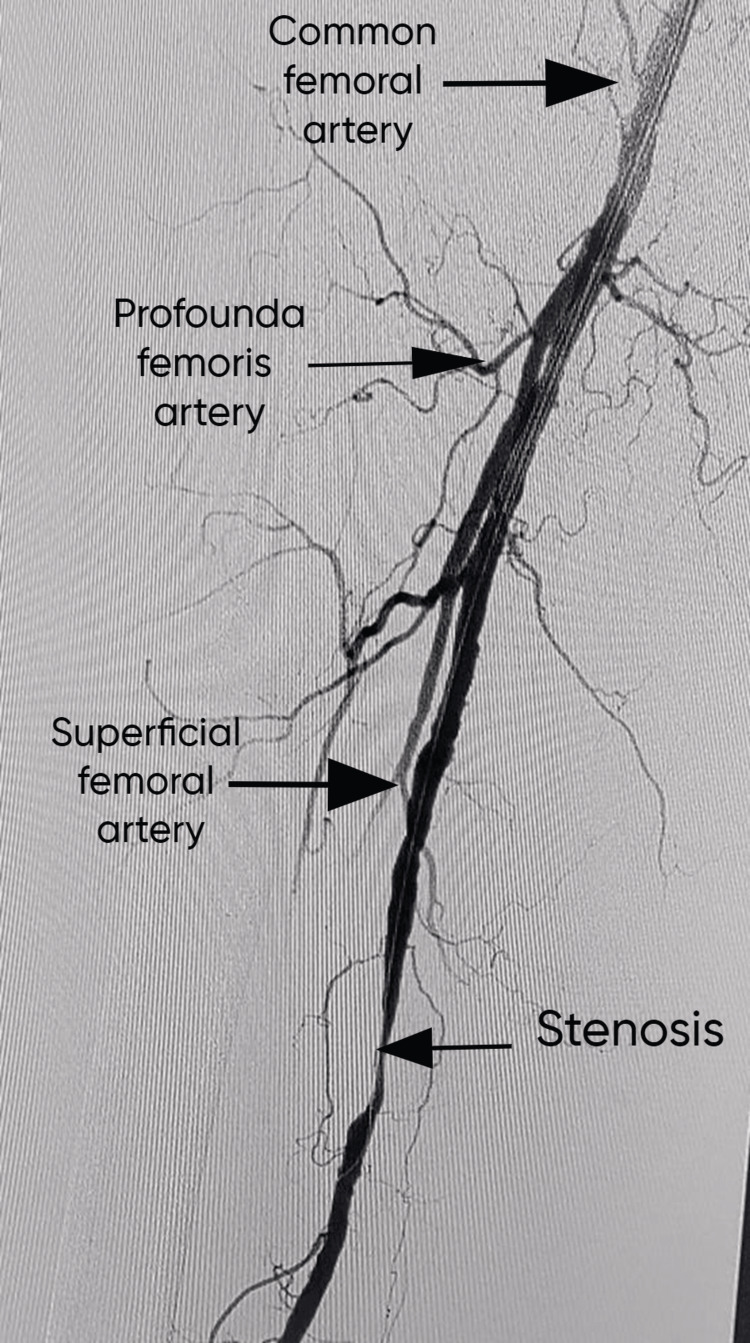
Stenosis or narrowed segment that defines PAD PAD: peripheral artery disease

The patient underwent a string of staged endovascular procedures that had been intended to repair each segment of the artery one at a time. The superficial femoral artery's proximal lesions were the main focus of the first stage of revascularization by employing modern endovascular methods, such as drug-eluting stent implantation and balloon angioplasty, and the superficial femoral artery flow was successfully restored. Improved arterial patency and flow dynamics were shown by subsequent angiography along with the removal of major stenoses. To maximize blood flow, to the distal artery segments sequential treatment were carried out using a similar strategy of balloon angioplasty and stent insertion. Figure 1 shows the stenosis or narrowed segment that defines PAD.

**Figure 2:**
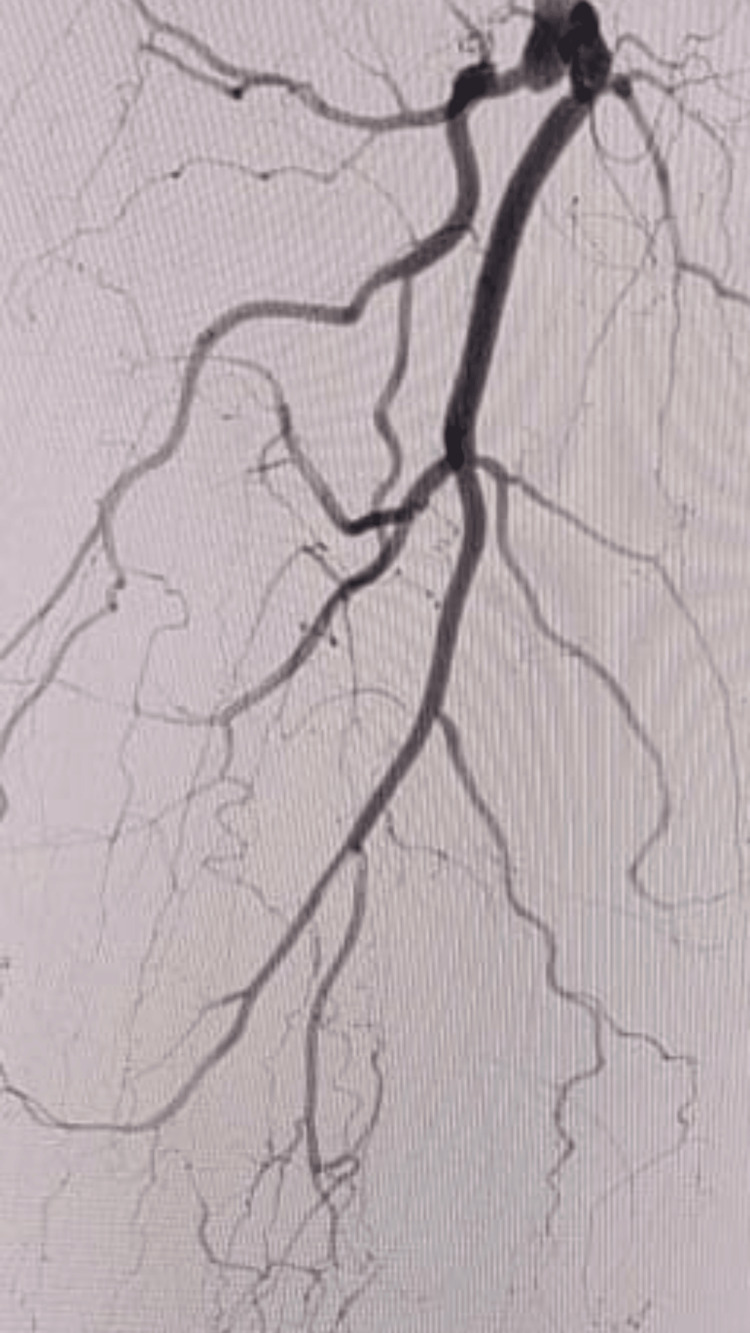
Complete thrombosis of the superficial femoral artery from the origin

Figure 2 shows complete thrombosis of the superficial femoral artery from the origin.

## Discussion

The common and debilitating condition referred to as PAD is caused by atherosclerotic blockage of the arteries supplying the limbs [8]. In the management of PAD, multilevel lesions, which involve both proximal and distal artery segments, present a special difficulty that frequently calls for a complete and specific strategy for revascularization [9]. A 67-year-old male patient came up with a history of hypertension and diabetes mellitus and initially complained of acute bilateral leg pain. Modal angioplasty was used to address the difficult multilevel PAD diagnosis. The management of multilevel PAD requires an understanding of the underlying arterial pathology and a multidisciplinary approach involving vascular surgeons, interventional cardiologists, and radiologists. To direct treatment decisions and improve procedural success, angiography is needed to determine the location extent and severity of arterial defects. The complex nature of the patient's vascular disease was shown in our case by diagnostic angiography, which displayed multilevel lesions affecting the superficial femoral, popliteal, and tibial arteries.

For our patient, the progressive angioplasty string of staged endovascular procedures that target each artery segment one at a time was decided to be the most effective method of action. The possibility to modify the treatment plan in response to intra-procedural findings, focused intervention, and full lesion assessment are just a few advantages provided by this method of treatment. Sequential angioplasty maximizes procedural success while lowering the risk of complications associated with major revascularization treatments by addressing each artery segment in turn [10]. Our patient had an endovascular procedure to treat the proximal lesions involving the superficial femoral artery during the first phase of revascularization by implementing advanced endovascular methods, such as drug-eluting stent implantation and balloon angioplasty, and the superficial femoral artery's flow was successfully restored. The lesions in the tibial and popliteal arteries were the focus of further procedures, which similarly resolved the substantial stenoses. Six-month follow-up angiography showed patent vessels free of restenosis, indicating the treatment plan's durability. In managing severe multilayer PAD, this case demonstrates the effectiveness of successive angioplasty in obtaining symptomatic alleviation while maintaining limb perfusion. More investigation is needed to evaluate the durability and long-term effects of sequential angioplasty in similar patient populations. This research has the potential to enhance clinical practice and outcomes for the treatment of PAD.

## Conclusions

In conclusion, managing multilevel lesions in complicated PAD needs a personalized, collaborative approach. The patient is suggested to follow regular physiotherapy sections and also to look after himself with a proper diet chart. We aim to reduce symptoms, enhance blood flow, and lower the risk of problems by following a customized treatment plan, lifestyle modifications, and frequent follow-up care. The success of the procedure and treatment also depends on the operator's skill and commitment. These procedures are time-consuming and thus need the patience of both patients and operators to see the expected results.
